# Technology-Enabled Reform in a Nontraditional Mental Health Service for Eating Disorders: Participatory Design Study

**DOI:** 10.2196/19532

**Published:** 2021-02-16

**Authors:** Alyssa Clare Milton, Ashlea Hambleton, Mitchell Dowling, Anna Elizabeth Roberts, Tracey Davenport, Ian Hickie

**Affiliations:** 1 University of Sydney Sydney Australia; 2 Innowell Sydney Australia

**Keywords:** eating disorders, body image, mental health, technology, co-design, participatory design, service reform, consumer engagement

## Abstract

**Background:**

The recent Australian National Agenda for Eating Disorders highlights the role technology can play in improving accessibility and service development through web-based prevention, early access pathways, self-help, and recovery assistance. However, engagement with the eating disorders community to co-design, build, and evaluate these much-needed technology solutions through participatory design processes has been lacking and, until recently, underresourced.

**Objective:**

This study aims to customize and configure a technology solution for a nontraditional (web-based, phone, email) mental health service that provides support for eating disorders and body image issues through the use of participatory design processes.

**Methods:**

Participants were recruited chiefly through the Butterfly National Helpline 1800 ED HOPE (Butterfly’s National Helpline), an Australian-wide helpline supporting anyone concerned by an eating disorder or body image issue. Participants included individuals with lived experience of eating disorders and body image issues, their supportive others (such as family, health professionals, support workers), and staff of the Butterfly Foundation. Participants took part in participatory design workshops, running up to four hours, which were held nationally in urban and regional locations. The workshop agenda followed an established process of discovery, evaluation, and prototyping. Workshop activities included open and prompted discussion, reviewing working prototypes, creating descriptive artifacts, and developing user journeys. Workshop artifacts were used in a knowledge translation process, which identified key learnings to inform user journeys, user personas, and the customization and configuration of the InnoWell Platform for Butterfly’s National Helpline. Further, key themes were identified using thematic techniques and coded in NVivo 12 software.

**Results:**

Six participatory design workshops were held, of which 45 participants took part. Participants highlighted that there is a critical need to address some of the barriers to care, particularly in regional and rural areas. The workshops highlighted seven overarching qualitative themes: identified barriers to care within the current system; need for people to be able to access the right care anywhere, anytime; recommendations for the technological solution (ie, InnoWell Platform features and functionality); need for communication, coordination, and integration of a technological solution embedded in Butterfly’s National Helpline; need to consider engagement and tone within the technological solution; identified challenges and areas to consider when implementing a technological solution in the Helpline; and potential outcomes of the technological solution embedded in the Helpline relating to system and service reform. Ultimately, this technology solution should ensure that the right care is provided to individuals the first time.

**Conclusions:**

Our findings highlight the value of actively engaging stakeholders in participatory design processes for the customization and configuration of new technologies. End users can highlight the critical areas of need, which can be used as a catalyst for reform through the implementation of these technologies in nontraditional services.

## Introduction

Over 1 million Australians currently have a clinically diagnosed eating disorder; however, only 25% of Australians with an eating disorder are known to the health system [1]. There has been a recent increase in government funding for the prevention, detection, assessment, and treatment of eating disorders, with a specific focus on using technology solutions to prevent the onset of eating disorders [2]. Across Australia in the last 12 months alone, there has been an Aus $200 million (US $155 million) investment in eating disorders. This includes Aus $70 million (US $54 million) for the establishment of seven residential eating disorder centers around Australia, Aus $4 million (US $3 million) for research translation, Aus $110 million (US $85 million) to fund dedicated Medicare services for eating disorders, and Aus $3 million (US $2 million) to Butterfly National Helpline 1800 ED HOPE (Butterfly’s National Helpline).

The Butterfly Foundation released a National Agenda for Eating Disorders with the aim of establishing a baseline for accessible evidence-based treatments for everyone affected by eating disorders in Australia [1]. Within this agenda, technology is identified as a facilitator of improving accessibility and national service development. The agenda identified the importance of developing existing digital services, such as Butterfly’s National Helpline to include web-based prevention, early access pathways, self-help, and recovery assistance. Further, technology solutions can address gaps in the continuum of care by extending existing eating disorder-specific web-based and telephone services to include self-help programmes for people with mild or subclinical bulimia nervosa and binge eating disorder and to provide recovery support services for individuals, carers, families, and friends.

In July 2018, The University of Sydney’s Brain and Mind Centre and the Butterfly Foundation partnered to engage the client base (individuals, supportive others [SO; eg, Carers, families, and friends] and other professionals affected by an eating disorder, disordered eating, or body image issue) and staff (counselors and service managers) of Butterfly’s National Helpline in participatory design workshops to explore how a web-based platform (ie, the InnoWell Platform) could be tailored to realize a technology solution for a nontraditional mental health service, such as Butterfly’s National Helpline. In brief, the Helpline is a free and confidential service that provides information, counseling, and treatment referral for eating disorders, disordered eating, body image, and related issues via telephone, web-based chat, or email. Butterfly’s National Helpline counselors are professionally trained and experienced in supporting those affected by an eating disorder—the individual who is struggling with their journey and their SO. The Helpline provides information, support, and guidance on treatment options as well as referral pathways on an as-needs basis, and is delivered as a brief intervention with no ongoing therapeutic engagement with those who contact the service. Commonly, the Helpline is the first contact point for an individual with concerns about their eating and body image and those that have not experienced any type of treatment to date [3].

Prior to engagement with the Butterfly Foundation, a partnership project called Project Synergy (Phase 1: 2014-2016) was undertaken by the Young and Well Cooperative Research Centre and The University of Sydney’s Brain and Mind Centre. Project Synergy (Phase 1: 2014-2016) was originally commissioned by the Australian Government Department of Health in 2014 (Aus $5.5 million [US $ 4.18 million]), with the broad aim of transforming the provision of mental health care across Australia by harnessing the potential of new and emerging technologies to reach all people, regardless of location, and provide them with access to timely and evidence-based treatment to improve their mental health and wellbeing [4]. Phase 1 of Project Synergy established a research and development (R&D) cycle that used participatory design methodologies to co-design, build, and evaluate a prototypic web-based platform. Project Synergy (Phase 2: 2017-2020) is another Australian Government Department of Health-funded initiative (Aus $30 million [US $ 22.81]), which is delivered by InnoWell Pty Ltd (a joint venture between the University of Sydney and PwC [Australia]) and aims to iterate the prototypic web-based prototype to the InnoWell Platform.

The InnoWell Platform links the integrated and interoperable resources (eg, apps, etools, web-based and in-clinic health services, most with data sharing functionality) to enhance service quality, track real-time health and social outcomes, and bring integrated, high-quality, and personalized service experiences to the individual seeking care. It can operate through existing health providers, such as Butterfly’s National Helpline, to promote access to high-quality and cost-effective mental health services.

Importantly, the goal of the InnoWell Platform is to offer immediate web-based assessment (all individuals complete a tailored self-report questionnaire) resulting in a personalized dashboard of results. The results provide individuals with an overall profile of their health and wellbeing (including mental health), which can be shared with their health professional (HP), other health care providers, and family members, among others (dependent on permission being granted by the individual). The platform utilizes staged care based on a transdiagnostic clinical staging model [5,6] to identify the extent of disease progression at a point in time. This enables the platform to match recommendations, including apps and etools as well as clinical interventions to an individual’s level of need.

At its core, the InnoWell Platform promotes person-centered health care and its principles highlight that individual clients of a service are equal partners in their health care. To that end, to promote transparency, individuals have access to all information that directly concerns them. Furthermore, all information is presented in plain language, and individuals are presented with sufficient information to understand all components of the Platform (for example. self-report questionnaire, dashboard of results, etc), with options for obtaining further information if desired. Critically, decisions about an individual’s care are made collaboratively with a HP or service, taking into account both clinical needs and personal preferences. The platform helps minimize variability in care provision between individual HPs and services by utilizing evidence and data rather than relying solely on clinical opinion, which can be variable and fallible. Finally, the platform is designed to maximize the use of resources and minimize duplication of services and wastage of time for all individuals.

A key feature of the platform is that it can be customized and configured to meet the needs of all end-users, including individuals and SO, HPs, service managers, and administrators. By engaging potential end-users through the iterative use of participatory design, the platform can be continuously developed to best meet the needs of a health care service.

Research has shown that using participatory design processes to co-design technology solutions allows for the active participation of all stakeholders and helps ensure that the end product meets the needs of its intended user base, improves usability, and increases engagement of all individuals [4,7]. Through the engagement of stakeholders in co-design, technology solutions to practical problems related to health care are generated as a means to effect reform [8]. Importantly, end-users (in this instance, all members of Butterfly’s National Helpline community) have the opportunity to actively co-design the technology solution in conjunction with researchers and product designers with the aim of developing a web-based clinical tool that is more likely to be engaging and effective for all users [7,9].

The aim of the current research was to actively engage individuals from Butterfly’s National Helpline community, via co-design, to collaboratively customize and configure the InnoWell Platform to enhance access to and service quality of Butterfly’s National Helpline.

## Methods

This research was approved by the University of Sydney Human Research Ethics Committee (Project number: 2018/041).

### Participants

Participants of the participatory design workshops (detailed below in the Participatory Design Workshops section) were part of Butterfly’s National Helpline community. This included individuals with a lived experience (LE) of eating disorders, disordered eating, body image and related issues, SOs, HPs (including Butterfly’s National Helpline counselors), service managers, and administrators.

To be eligible to participate in the face-to-face workshops, members of the community (described above) had to be aged 15 years and older and proficient in English.

### Recruitment Strategy

The recruitment strategy included the distribution of digital and nondigital postcards and A3/A4 posters with information about the participatory design workshops in the lead up to each scheduled workshop in each of the six locations. Specifically, participants were recruited via Butterfly National Helpline management, Butterfly Recovery Support Service group facilitators, targeted emails to stakeholders, and advertisements in relevant services (for example. *headspace* Darwin). To avoid any perceived coercion, recruitment was passive such that a potential participant needed to contact the Research Project Manager who, only upon a potential participant’s request, then forwarded the study information sheet and participant consent form.

In line with Project Synergy’s recruitment process (Reported in the study by LaMonica et al [10]), all participants were provided with detailed information about the research prior to attending a participatory design workshop. Potential participants completed a brief Screener Survey to determine eligibility for the research. Once eligibility was confirmed, potential participants were given the opportunity to provide consent. At the beginning of each workshop, the facilitators provided a second opportunity for participants to ask questions and clarify details of the research prior to providing their written informed consent. Participants were reminded that participation was entirely voluntary, and that if they agreed to participate, they could withdraw their consent at any time without being required to provide any reasons and with no impact on their relationship with The Butterfly Foundation, Butterfly’s National Helpline, The University of Sydney’s Brain and Mind Centre or InnoWell Pty Ltd.

### Participatory Design Workshops

**Figure 1:**
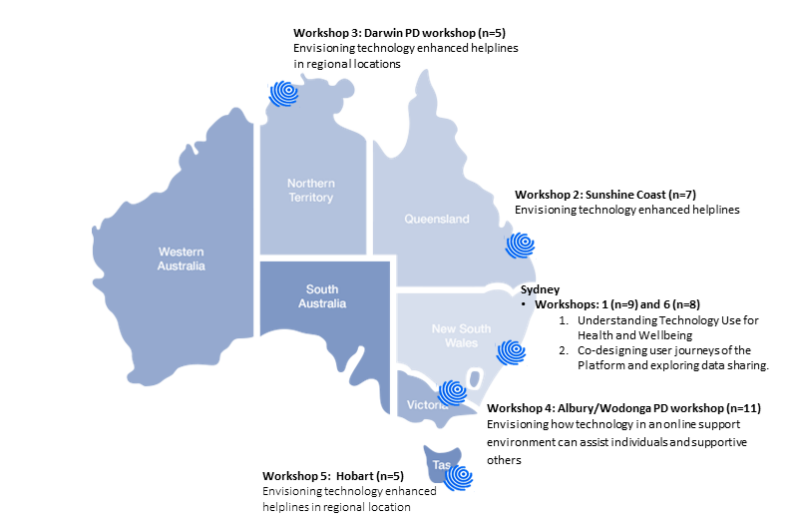
Workshop location, sample size, and focus. PD: participatory design.

Participatory design workshops were run face-to-face across Australia in diverse urban and regional centers. Final locations were determined through a collaborative discussion between the Butterfly Foundation and the researchers. Locations were selected in areas where the Butterfly Foundation had a physical presence and local partnerships, and there was representation from at least one major capital city, two regional centers, and one location that was regional or otherwise isolated from eating disorder services. Six participatory design workshop locations were selected, which included two held in Sydney (major capital city), the Sunshine Coast (major city; inner regional center), Albury Wodonga (inner regional center), Darwin (outer regional), and Hobart (inner regional center). Each workshop lasted for up to four hours. As per the Project Synergy R&D Cycle [4], the series of workshops were conducted rapidly (between February and May 2019) to maintain the momentum of idea creation, continuing until theme saturation had been reached. In addition, key follow up themes based on the ideas generated in previous workshops were explored as a main focus in later workshops. This was carried out to ensure that ideas concerning these key topics had been fully saturated (Figure 1 for main focus areas). All workshops were coordinated by at least two facilitators, one of whom was a mental HP whose role was to respond to any participant concerns or distress as a result of the subject matter. A scribe was present to take detailed hand-written notes and quotes throughout the workshop. An important component of the face-to-face participatory design workshops was that no technology was used, as research has shown that this approach results in the generation of more ideas and design solutions [11].

Each participatory design workshop was designed to actively engage participants in interactive discussions about how to co-design potential technology solutions for Butterfly’s National Helpline community and how the InnoWell Platform could be customized and configured to enhance service provision. The workshop agendas were collaboratively refined by the joint research management team, comprising researchers, HPs, and members of the Helpline community, to determine how best to discover how the technology solution might enhance or reform the service. As shown in Table 1, the workshops used a three-phased approach of discovery, evaluation, and prototyping [4]. Several specific focus areas were explored in depth in the discovery phase, which were conducted using a semistructured topic guide. Focus areas included understanding: the role of the Helpline compared to other types of services and supports (face-to-face, telemedicine, apps, and etools); how technology is used for health and wellbeing; how technology can assist individuals and SO using the Helpline; and understanding issues arising in both urban and regional areas. In the evaluation stage, a variety of methods were employed, including a review of working prototypes (wireframes) and gaining feedback on ideas generated in previous workshops through prompted discussion. In the prototyping stage methods included creation of descriptive artifacts and mock-ups, and group-based and individual development of user personas (hypothetical typical end users of the InnoWell Platform including their profile, user group, background and history, current situation, goals and motivations, frustrations, and challenges) and user journeys (series of steps illustrating how end users might interact with the Platform). As highlighted in other research [10], user journeys assist in understanding user behavior, identifying other potential areas of platform functionality for future development, defining both the taxonomy and interface, and feedback into a number of technology building activities including information architecture and sitemaps, the development of wireframes, and functional specifications.

**Table 1:** Participatory design workshop stages, description and focus.

Stage	Description	Methods	
		Type	Focus
Discovery	Open and prompted discussion to explore participant practices, goals, values and needs within their group and region.	Qualitative exploration using semistructured interview guides	General views based on individual’s goals, needs and values relating to the Helpline and technology The role of the Helpline compared to other types of services and supports (face-to-face, telemedicine, apps and etools) The use of technology for health and wellbeing The use of technology to assist individuals and supportive others using the Helpline Understanding issues arising in both urban and regional areas
Evaluation	Participants explore and evaluate current resources focusing on their strengths and weaknesses.	Group-based and individual review work	Review of working prototypes (wireframes) Feedback on ideas generated in previous workshops through prompted discussion
Prototyping	Brainstorming with participants as they suggest ideas, sketch concepts, and envision the technology solution.	Group-based and individual development work	Descriptive artifacts and mock-ups User personas (hypothetical typical end users of the InnoWell Platform including their profile, user group, background and history, current situation, goals and motivations, frustrations and challenges) User journeys (series of steps illustrating how end users might interact with the Platform)

At the conclusion of the workshops, individuals with LE and SO received a web-based Aus $50 (US $ 38.01) voucher of their choice (Woolworths, Big W, Caltex, Coles, Target, Kmart, JB Hi FI, or Prepaid Mobile Recharge) as reimbursement for their time. HP and service provider participants received reimbursement only if research was conducted outside of standard working hours.

### Data Analysis

As described in other research [10], at the conclusion of each workshop, the notes and quotes taken by the scribe, combined with any facilitator notes, were transcribed into a report documenting the participant background (ie, participant type such as HP or individual with lived experience) as well as the content of the discussion relative to the agenda. These reports, in combination with the visual artifacts collected during the participatory design workshops (nonidentified and presented as aggregate data to ensure confidentiality) were analyzed by the knowledge translation team, which is a group of people who represent various stakeholder backgrounds and can implement research findings into practice. The team for this research included LE representatives, mental HPs, researchers, and a co-design program manager who had experience in product management. The knowledge translation processes identified themes and key learnings to inform the customization and configuration of the InnoWell Platform for Butterfly’s National Helpline. In brief, knowledge translation is an interactive process of synthesizing, exchanging, and applying knowledge [12]. With the ultimate goal being that the research findings are translated into clinical practice, organizational management, technology development, and policy reform [12].

In addition, all workshops notes, artifacts, and reports were anonymized and reviewed by three researchers (AM, AH, AR) to develop a coding framework outlining all key concepts. Data were coded in NVivo 12 software using this framework. Interpretation of the data followed established thematic techniques [13], which involved an iterative process of reading, coding, exploring the pattern and content of coded data, reflection, and discussion. Similarities and differences in opinion were examined, and differences dealt with through discussion to reach consensus. This qualitative analysis strategy has been utilized in the past literature [14-16].

When presented in the Results section, the analyzed data sources are categorized into three key areas: (1) *Notes*, which are the field notes taken by the scribe during the workshops; (2) *Prototype*, which comprises the visual artifacts developed by participants; and (3) *Report*, which is the participatory design report collated immediately after the workshop by the facilitators and the scribe that summarized the workshop findings. Further, the participant source presented in the results section in the parentheses after a quote included: (1) participants with LE of eating disorders, disordered eating, body image, and related issues; (2) participants who were HPs and identified as having a LE; (3) HPs who were clinicians but may also perform an administrative or service manager role (HP); (4) SO, or participant background not specified (PBNS). The analyzed data are presented as results in inverted commas. Data derived from the notes and artifacts are direct quotes from participants, whereas the data presented in the report may be a summary of the findings paraphrasing the participants.

## Results

### Demographics

Six participatory design workshops were held in diverse urban and regional centers across Australia, including Sydney (Workshop 1: n=9), the Sunshine Coast (Workshop 2: n=7), Albury Wodonga (Workshop 3: n=11), Darwin (Workshop 4: n=5), Hobart (Workshop 5: n=5), and Sydney (Workshop 6: n=8). In total, 45 participants attended the workshops. Participants included people from Butterfly’s National Helpline community, 13 of whom (29%) were identified as having a LE of eating disorders, disordered eating, body image, and related issues; 4 (9%) were HPs with lived experience; 21 (47%) were HPs; and 7 (16%) were SOs. Figure 1 shows the location, main focus, and participation rate across each workshop. No participants expressed concern or experienced any distress in any of the workshops.

**Figure 2:**
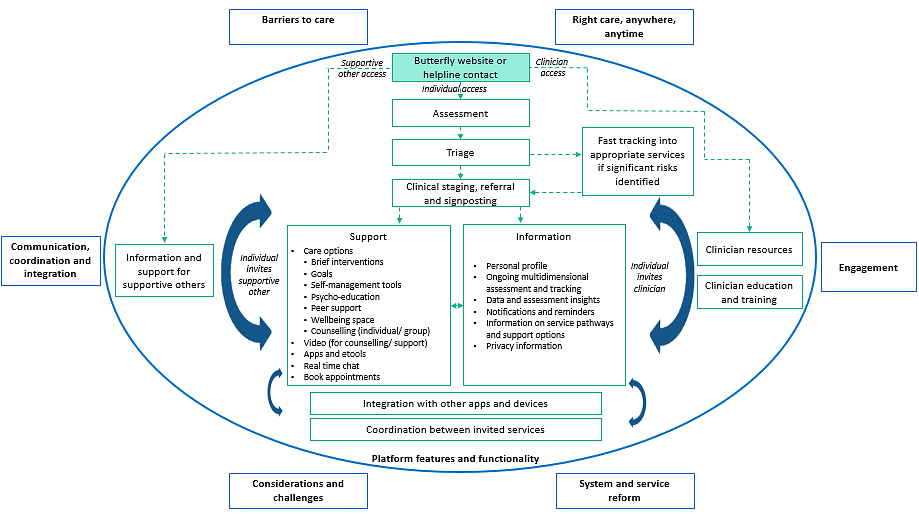
Overarching participatory design themes.

The results presented in Figure 2 highlight the seven overarching themes identified by participants during participatory design workshops. Within the oval, the co-designed overview of the InnoWell Platform is represented, whereas outside the oval, general themes from the top to the bottom of the figure reflect before, during, and after using the co-designed InnoWell Platform. Specifically, the themes presented above the oval highlight participants’ ideas concerning accessing care, which included the identified barriers to care within the current system (Theme 1: barriers to care) and the need for people to be able to access the right care, anywhere, anytime (Theme 2: Right care, anywhere, anytime). The theme within the oval comprises recommendations for the actual technological solution that was co-designed by participants, ultimately representing the InnoWell Platform features and functionality (Theme 3: features and functionality). The outside central themes relate to general recommendations if an individual uses the InnoWell Platform. These themes relay the need for communication, coordination, and integration of a technological solution when embedded in Butterfly’s National Helpline (Theme 4: Communication, coordination, and integration) and the need to consider engagement and tone within the technological solution (Theme 5: Engagement). Finally, the lowermost themes relate to the identified challenges and areas to consider when implementing a technological solution in the Helpline (Theme 6: Considerations and challenges) and the potential outcomes of the technological solution embedded in the Helpline relating to system and service reform (Theme 7: System and service reform). Each theme is discussed in detail in the following results.

### Barriers to Care

Difficulties accessing vital psychological or medical care were reported in all participatory design workshops across all participant groups (76 references). However, there were some notable differences for regional populations, which described factors associated with regionality as the most frequently reported *barrier to care*. Access for those living in regional areas that border other regional towns can also be complicated by political and health district boundaries, “...no trained clinicians in Albury, but cannot see the clinicians in Wodonga. Costs more to treat an ED [Eating Disorder] in NSW” (Notes, SO). One solution is the use of telemedicine; however, it was noted that the financial burden of care reduces the feasibility of this solution. For example, one SO highlighted that there is “...not much availability, just as expensive as seeing someone in person” (Darwin, Notes). A *lack of quality services* was described “One person claims to have expertise, other than that there’s nobody up here to talk to.” (Notes, SO). Unique to living in a regional area, the issue of *anonymity* was raised, “Anonymity is very important in a country town due to the stigma of help-seeking” (Report, SO). The consequences of the illness such as the beliefs of being “not sick enough” and “not deserving help” were also highlighted as *barriers to care* (Workshop 1, Sydney, Notes, HP).

### Right Care, Anywhere, Anytime

The theme *right care, anywhere, anytime* was referenced in all six workshops (83 references). This theme highlighted that technology could “reduce wait times” (Prototyping, HP) by “speed[ing] up getting access to the right support” (Notes, Participant with LE) where there is “no delay in response or engagement of support...[as individuals have] often waited too long to make contact” (Prototype, PBNS). Ultimately, the InnoWell Platform could provide “...broader ways of access—virtual, Skype, irobot for excess calls so the phone is answered [by the Helpline]” (Prototype, PBNS). One HP felt that the Platform could function as “a stepping stone into the service—[where the] Platform is the first point of contact” (Prototyping, HP), which might be accessed via The Butterfly Foundation’s website (Prototype, PBNS).

**Figure 3:**
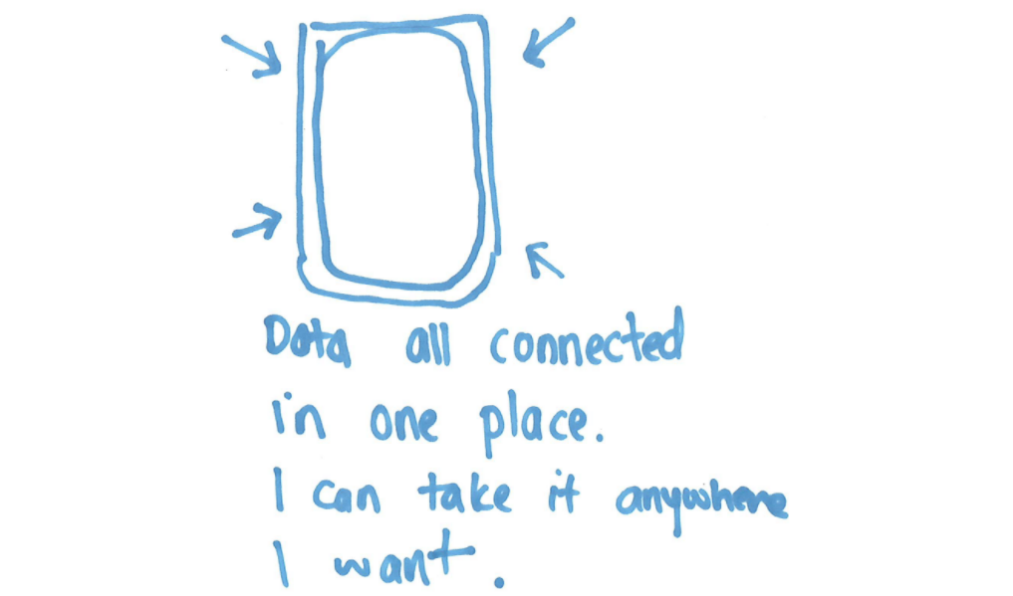
Artifact showing an individual’s data connected all in one place (Workshop 2, Sunshine Coast, Prototype, Lived Experience).

Ultimately, the technology could be used to “meet people where they are at” (Notes, HP) and, as illustrated in Figure 3, be able to do this at a time and place that suits their needs. For example, it was emphasized that “Being able to connect (with someone) outside of regular session times (would be important)” (Notes, SO). Additionally, the technology was viewed as needing to be free, easy to use, *respond in real time* and facilitate “Finding the expertise—wherever they are in Australia” (Notes, SO), which may be particularly beneficial for regional and rural individuals.

### Features and Functionality

The flow of *features and functionalities* that could support an end user through an imagined web-based platform, while complementing Butterfly’s National Helpline were discussed the most frequently (306 references) and are presented in detail within the oval displayed in Figure 2.

As a first step, end-users entering the InnoWell Platform would have the option of having their needs assessed. Assessment results would enable the Platform to triage the end user. If the end user presented with significant risks—such as extreme eating disorder concerns or suicidal thoughts and behaviors—additional support and fast tracking into appropriate services would be provided via the technology. If the end user did not require this, the platform would use the individual’s clinical stage to give personalized referral suggestions, signpost to support, and provide the end user with relevant information. Ultimately, this was envisaged as “an automated, staging, triage platform” (Notes, HP) which “Provides support and resources based on different stages/levels of severity and distress” (Report, PBNS).

*Support features* included providing brief interventions (ie, care options) such as “motivational interviewing sessions” or “guided CBT (cognitive behavioral therapy)” (Notes, HP), self-management tools, peer support, goal setting, and wellbeing space. For example, one SO in Darwin suggested that the InnoWell Platform could create a space where the individual could “list the positives or their goals (eg, passions for life and hobbies). The things that will motivate them to recover” (Notes). These care options would be supported through the use of apps and etools, video counseling as “People respond so much better on the video, phone is not enough” (Notes, HP), real time chat “with someone who knows your case and can respond in real time” (Notes, SO), and appointment booking as “Sometimes it’s hard for clients to make an appointment” (Notes, HP). The information features and functionality would include a personal profile of the end user, psychoeducation, information on service pathways, an easy-to-read privacy statement, and data and assessment insights presented on the platform’s dashboard of results, which provides the opportunity to complete ongoing multidimensional assessment.

**Figure 4:**
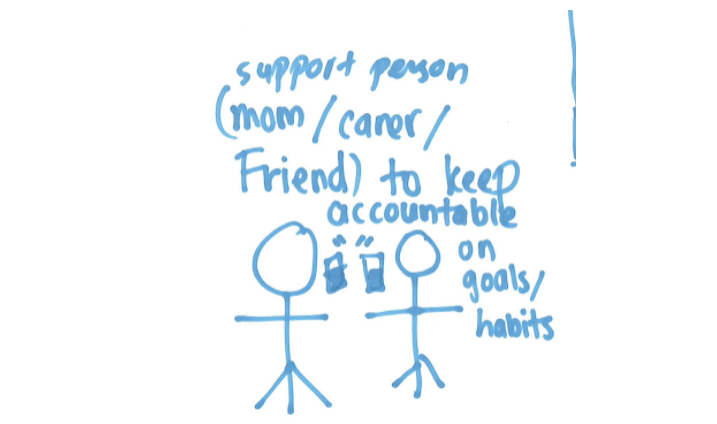
Artifact showing data sharing with a supportive other (Workshop 2, Sunshine Coast, Prototype, Lived Experience).

The InnoWell Platform was also seen as a resource and clinical tool for HPs and SOs. Via the Platform, HPs would have access to education and training for eating disorders and body image concerns alongside resources that could be tailored by region. SOs would also have access to tailored information (such as understanding the service pathways and available resources) and support (such as peer-support and strategies for self-care). HPs and SOs could access the platform in two ways. The first is via Butterfly’s National Helpline to access the above-mentioned resources. The second access pathway was via invitation from people with LE who were using the Platform themselves. This would allow HPs and SOs to provide support and be “on the same page” (Report, PBNS) as the individual they were supporting. One HP highlighted that “Bringing in a support person is key to being able to help the person” (Notes, HP). Figure 4 provides an example of how one participant with LE prototyped what this support via data sharing might look like. Importantly, it was emphasized that end users on the Platform were in control of linking and sharing their data with HPs and SOs.

Another feature included end users being able to integrate the InnoWell Platform with other apps, devices, or medical records, and the services they were in contact with. This meant that all data and all nominated people and services involved in support of the end user were on the same page. This connection with multiple services was illustrated in an envisaged user journey, where:

...a young female teenager who is in the midst of a transition period (changing schools). She had a background of abuse. She contacts Butterfly’s National Helpline, which connects her with the InnoWell Platform, where she has access to online resources. Through the Platform, she is connected with services. She completed a further assessment. With her health professional, she establishes goals and agrees on care options.

Report

**Figure 5:**
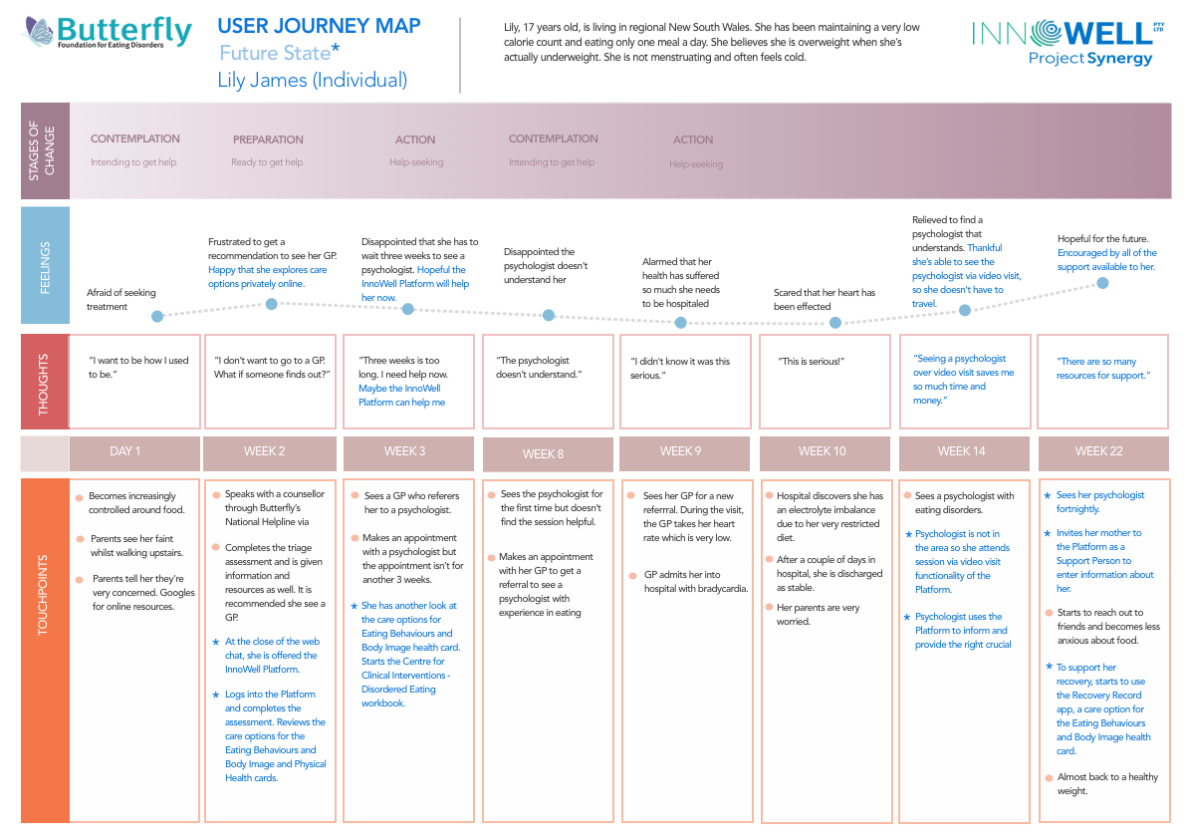
User journey of an individual with lived experience of an eating disorder.

Additional illustrative user journeys detailing how the imagined Platform might be used by end users were developed further by the knowledge translation team. These user journeys were based on combined participatory design workshop artifacts developed by participants in the prototyping stage of the workshops. Figure 5 presents an imagined journey of an individual with LE of eating disorders (a support person’s journey is presented in Multimedia Appendix 1).

### Communication, Co-Ordination and Integration

Theme *communication, coordination, and integration* were frequently referenced in all six workshops (109 references). This theme highlighted the importance of the InnoWell Platform being able to assist with a collaborative care approach, where decisions were made by the individual with support from their “team” (Prototype, PBNS). This would involve *care coordination* via a multidisciplinary team approach that included allied health, “psychologists, dieticians, GPs” (Notes, PBNS), other services involved in care, and the SO where possible (as detailed above in *Platform Features and Functionality*).

A concern with the current helpline support structure was that it was delivered as a one-off session of counseling. One SO highlighted that “You need the same person, you need trust, and you need ongoing connection and ongoing care. You just need to use the existing technology. A single session is not helpful.” (Notes). Ultimately, maintaining *continuity of care* for an individual was viewed as paramount. Technology was seen as potentially helping this process, as it provided a place for case conceptualization and a communication pathway. For example, a process was suggested were technology could facilitate “a case review and follow up of the caller” (Notes, PBNS) after contact with the Helpline. Another HP highlighted that “tech allows services to talk to each other” (Notes, HP), whilst an individual with LE after being shown the InnoWell Platform’s dashboard of results for evaluative purposes in a later workshop stated it “Puts all [the information] in one spot which is good” and an individual using the Platform “Can show all different parties” (Notes, PBNS).

### Engagement

The user experience of technology-based psychological tools and resources is largely shaped by *engagement* (150 references). Technology offers many solutions to mental health and wellbeing; however, the *need for human touch* was discussed as a priority at all workshops. The “importance of maintaining language so still a human touch, not moving too much towards bot” (Notes, HP) was raised, as were the qualities that are unique to humans: “I don’t know how it replaces a hug and compassion. I do not know how tech can do that” (Notes, SO).

The impact of *language* was widely recognized as crucial to the engagement with, and tone of, the InnoWell Platform. In one workshop, it was noted “Keep language simple, broad and recovery focused. For example, include statistics of people who seek help and recovery success rates, create hope” (Report, LE). Recovery-focused language entails motivational and hope messages. Engagement would be increased by *personalized* care. Personalization includes providing choice to the users of the Helpline, “Empower consumers with choice. So much variety of what is available enables them to discern the information and choose what they want, when they want” (Report, PBNS). Difficulties in navigating credible and trustworthy information were described at all of the workshops. The “Importance of trustworthy information and resources, as well as information not being too overwhelming” (Report, PBNS) was described, otherwise “Information can be confusing if too much or not linked to trusted resources” (Notes, PBNS). Further to information provision, trust was described as crucial to care “You need the same person, you need trust, you need ongoing connection and ongoing care” (Notes, SO).

### Considerations and Challenges

*Considerations and challenges* in using a technology solution (ie, The InnoWell Platform) for the Helpline were discussed in all six workshops (109 references), particularly in the Hobart and Darwin workshops.

The most frequently referenced consideration is related to the set-up of the *InnoWell Platform and infrastructure.* Chiefly in Hobart, Darwin, and the second Sydney workshop, there were *considerations and challenges* raised about *assessment, tracking, and data insights*. In terms of *assessment*, a SO highlighted that a full assessment was “too much work” (Notes, SO) and “a screener rather than a full assessment would be helpful, as it would screen the person for any major issues yet not be as time consuming” (Report, SO). Other key suggestions for the assessment were that a Butterfly counselor could “offer to start the assessment together” (Report, PBNS) with the individuals and provide breaks or chunk the assessment.

It was highlighted that in the eating disorder space, *tracking* though ongoing assessment could be problematic as “Scores could engage perfectionism” and “progress [is] hard to measure” (Notes, HP). One individual with LE highlighted that an “app actually fueled [my] eating disorder more, because [of] keeping track of things, and people with eating disorders have a desire to control their surroundings” (Notes, LE). Potential solutions to the tracking issue was that the InnoWell Platform could “Provide an option to ‘opt-in’ for tracking during informed consent”, as well as “Communicate tracking progress/graph using the recovery model so the individual does not lose hope” and “Frame the Platform as a ‘pal’ (more friendly), [as it] lets you know how you are doing, and asks check-in questions” (Report, PBNS).

**Figure 6:**
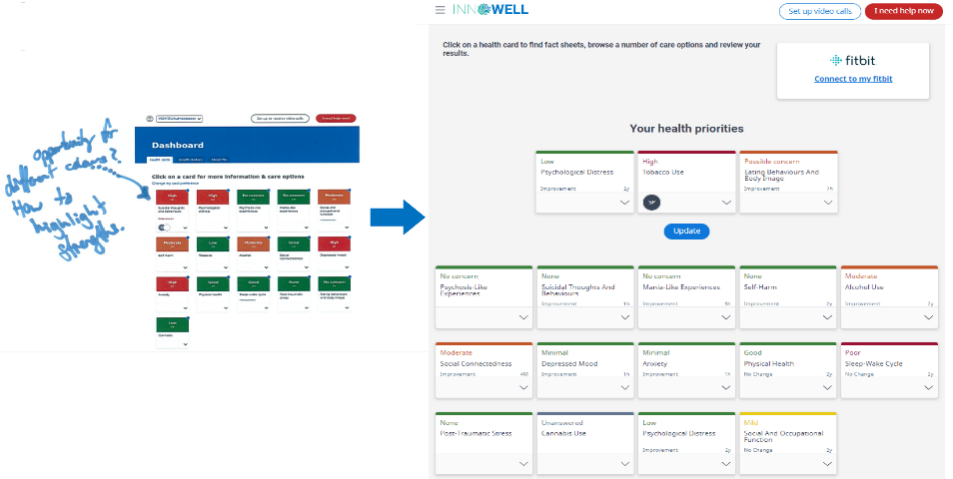
Illustrative example of how user feedback during participatory design workshops have informed changes to the InnoWell Platform.

The third consideration relates to the need to present data insights (on a dashboard of results) in a nonconfronting and nonstigmatizing manner. For example, one SO stated that “The dashboard with a ‘wall of red’ would be problematic for someone who already thinks they are a piece of shit. It just confirms you have got so many problems.” (Notes, SO). A HP in the same workshop suggested that data insights should present small amounts of information at one time “...to prevent visual overload” and that “...resilience and strengths need to be included to help the person feel like they have some positive aspects” In line with the *engagement* theme discussed above, an individual with LE in a later workshop highlighted that “Language used like ‘Psychological Distress’ [is] too confronting/strong for youth and may reinforce stigma” (Notes, LE). How feedback has been used to make changes to the InnoWell Platform to use more strength-based language and color schemes is presented in Figure 6.

When an individual using the InnoWell Platform was presented with possible care options, there were multiple *resource and information considerations* highlighted by workshop participants. These related to ensuring the Platform offered existing, credible, fact-based information, which was tailored to the individuals by need and geography, and this information was actually helpful. For example, one individual with LE highlighted that “The worst thing to see is ‘contact GP’. This is usually the first option but not helpful” (Notes, LE). There was a need to gather “...feedback on what works/what didn’t work” in terms of the resources provided to the individual. Further, it was important for people to feel that they were not just redirected to resources, but rather supported in the options provided. A SO highlighted “it is impersonal to direct someone to another place” (Notes, SO).

Three main *service mechanisms* are highlighted for consideration. This included ensuring that there were mechanisms to manage risk well by “...above all, do[ing] no harm” (Notes, HP), mechanisms to deal with feedback, and the need for the InnoWell Platform to operate within defined boundaries of the service. One HP in a manger position emphasized that the technology should “...focus on continually maintaining [the service] KPIs first” (Prototype, HP).

*Anonymity, privacy, consent, and transparency* were also important considerations. The option for people to maintain their anonymity was viewed as one of the benefits of using Butterfly’s National Helpline. This benefit was also seen to carry over to the InnoWell Platform. For example, one HP highlighted that “...being online takes away the shame and is anonymous” (Notes, HP). As highlighted in the *barriers to care,* this was viewed as particularly important in rural and some regional settings, with one SO illustrating this through their statement “What applies in urban Darwin does not apply in rural communities. There is no privacy. So if you go walk into a service, everyone will know your entire mental health history.” (Notes, SO).

Another key consideration related to the need for the InnoWell Platform to have the capacity to maintain confidentiality. One HP stated: “It’s about one person holding the body of information. A database that holds confidentiality and can be stored securely.” (Notes, HP). Furthermore, the consent process must be transparent. People with LE emphasized that “Individuals should be aware of what data is shared or escalated to the service. Ensure consent for this is provided” (Report, LE).

Considerations relating to an *individual’s needs* were highlighted in four of the six workshops. This theme emphasized the need for the InnoWell Platform to be able to support individuals with complex presenting issues, those who might be resistant to support via technology as well as those of particular socio-demographics such as age and culture.

Further, it was emphasized in two workshops that the InnoWell Platform had to consider the individual’s needs if a hypothetical situation arose where multiple viewpoints were included on the Platform, such as an individual with LE and their carer, but these views did not match. For example, a HP highlighted that the “...dissonance in the assessment results (eg, between carer and individual)” (Notes, HP) could be used as an opportunity for discussion. However, the individual’s preferences needed to be put first, so as to address the issue raised by another HP in the same workshop, who stated that often “...carers want all [the] info, but not all individuals with LE may be wanting [a] carer perspective.”

### System and Service Reform

Potential future outcomes concerning the use of technology solutions, such as the InnoWell Platform, within eating disorder services generally related to the theme *system and service reform,* which was raised by participants in all workshops, irrespective of their background (132 references). This is related to the potential of technology to facilitate improvements in a *service’s capacity* and the *clinical care* that could be provided.

In terms of *service capacity*, many potential outcomes are related to improvements in access to services. In one workshop, it was noted that it was “...possible that needs can be met without connecting face to face, with a clinician” (Report, PBNS). This was extended further in another workshop that imagined a scenario where technology could make wait-times more purposeful by “...re-direct[ing] them to resources whilst waiting” (Notes, HP with LE). In Darwin, a SO envisioned that technology could ensure that there was “...no delay in response or engagement support” (Notes, SO). It was envisioned that technology could make services “accessible to all” (Prototype, PBNS), particularly specific groups such as people living in regional and rural/remote areas and people who identify as “English as a second language users, males and lesbian, gay, bisexual, transgender, queer, intersex or asexual community groups” (Prototype, LE, and HP). Technology also had the capacity to improve access by “Reduce[ing] stigma and create[ing] pathways to care and support” (Report, PBNS), particularly as “Being online provides anonymity and removes stigma” (Report, PBNS).

The *clinical care* subtheme relating to *system and service reform* was referred to by participants in all workshops. This theme focused on five key areas where participants felt that technology could improve current care. The most frequently referenced area related to technology facilitates better *care coordination* (which is discussed in depth in the *communication, coordination, and integration* section). Technology could also meet care-related service gaps at follow-up as “Active efforts to follow up and transfer information is lacking” (Report, PBNS).

The next most referenced area related to improving the matching of interventions to the individual’s stage of illness, which we termed *clinical staging*—this was brought up as a potential *system and service reform* outcome in five of the six workshops. In the first workshop, it was noted that technology “Can support a staged approach to care, at early stages people might just want information and to learn about skills and resources via apps and websites” (Report, PBNS). A participant with LE highlighted that it would be ideal if the technology could be used “...at any stage of the journey” (Notes, LE).

*Continuity of care* as a potential *system and service reform* outcome was referenced in all six workshops. For example, one HP highlighted that technology could address some key issues around the current “...lack of flexibility to find the right care and have continuity of care” (Notes, HP), with an individual with LE in the same workshop extending this line of thinking by highlighting that often people accessing services “...have to keep telling the story over and over again” (Notes, LE). Data sharing through technology allows individuals to access and show others’ useful information, such as their history, current situation, support networks, and plans from one location. One HP exemplified this through their comment: “It’s about one person holding the body of information” (Notes, HP). Through technology, it was envisioned that people would have a greater capacity to engage in *self-management.* It could “...help people help themselves” (Prototype, HP).

## Discussion

### Principal Findings

The use of participatory design processes has enabled a collaborative approach to the customization and configuration of the InnoWell Platform for Butterfly’s National Helpline. This included a relatively large sample (n=45), compared to other participatory design research [16] with participants with a range of backgrounds, including individuals with lived experience, SOs, HPs, service managers and administrators, and those with mixed backgrounds. The ultimate aim of this process was to build a technology solution through prototyping that would enhance both access to and quality of care delivered through a nontraditional mental health service.

Participants felt that technology could enhance services’ accessibility (ie, provide the *right care, anywhere, anytime*) and reduce the *barriers to care*, which is a finding in line with other qualitative and participatory design research in the mental health field [17,18]. Of particular importance in the current research, engagement in this participatory design process spanned both urban and regional areas. It is clear from the findings, particularly within the *barriers to care* theme, that current service provision for eating disorders is critically lacking in regional (as well as rural and remote) areas. Research has shown that in Australia, people living in regional, rural, and remote areas are likely to experience persistent disadvantages [19,20], and disadvantaged communities experience considerable social and health inequalities [20-22]. In mental health, disadvantages based on regionality are often attributed to poor access to primary and acute care services, insufficient numbers of mental health services and HPs, cost of receiving care, distance required to travel to access care, concerns about stigma, cultural barriers relating to service access, and a general reluctance to seek help in these communities [23,24]. These concerns are also highly relevant to the eating disorder community residing in regional and rural areas, as they were emphasized within our participatory design workshops as fundamental *barriers to care*.

Although technology alone cannot solve all these identified issues for regional and rural communities, participants highlighted that Butterfly’s National Helpline augmenting and reforming its service using a technology solution could meet this burgeoning need to some extent. This is highlighted in the *system and service reform theme.* Further, the example user journey of a young woman with a LE of an eating disorder living in regional Australia highlights just how this might be done, using multiple forms of technology including the Helpline’s webchat, the InnoWell Platform, and quality-assured apps (which may be eating disorder focused or support other areas of an individual’s health and wellbeing) in conjunction with support from SOs and traditional services when they are available.

The remaining qualitative themes generated by participants in the discovery, evaluation, and prototyping stages of the workshop included communication, coordination and integration, engagement, features and functionality, and considerations and challenges. The ideas highlighted within these themes are also commonly cited in mental health focused on qualitative and participatory design research. This includes the importance of ensuring web-based tools and information are designed in such a way that they allow ease of navigation and comprehension in plain language [10,25,26]; maintain privacy and confidentiality [17,25,27,28]; ensure the information is reliable and accurate [25]; do not result in adverse impacts on the individual through use of the technology [26]; empower the individual and give them control and choice in their mental health care [10,17,25,28]; improving continuity of care within and between services [10]; support the information exchange between the clinician and the individual [10,25,26] through features and functionality such as access to assessment, progress monitoring and data tracking, data visualization, brief interventions, treatments, and reminders [10,26-28].

The InnoWell Platform is a clinical tool for clinicians (in this case, Butterfly’s National Helpline counselors) for use in providing care. In the participatory design workshops, participants co-designed this clinical tool to support not only individuals accessing care via Butterfly’s National Helpline, but also SO and HPs that support them (or are also supported by the Helpline). How this technology solution, which participants co-created in the prototyping stage, sits within the context of the Helpline is shown in Figure 2 and described in depth under *features and functionality*. The solution aims to promote person-centered care, which is collaborative, personalized, coordinated, and maintains continuity; to provide early intervention and support based on an individual’s clinical stage; to promote self‐management through the provision of information and support-related care options; and to provide key insights based on ongoing use of the Platform’s assessment. These features highlighted by participants in this study critically align with our other research on youth mental health [4,29,30], veteran populations [10], alongside other e-health solutions [31], which ultimately demonstrates the generalizability of some of these core components across both traditional and nontraditional services across the mental health sector, including eating disorder services. Ultimately, the co-designed solution adds value by demonstrating how technology can enhance a helpline service for multiple end-users with differing backgrounds. This is done by working collaboratively with end users to fully understand their needs, goals, and current practices, the context of the current support system, the challenges that may be faced when using the InnoWell Platform and how these might be addressed.

### Future Directions

In terms of potential service reform outcomes, the technology solution proposed by all end users had the potential to help services meet some of the key performance indicators for public mental healthcare. As outlined by Lauriks and colleagues [32], these include ensuring clinical safety, accessibility and equity, effectiveness and outcomes, acceptability and satisfaction, efficiency, expenditure and cost, appropriateness, continuity and coordination, and workforce competence and capability [4]. These indicators will form part of our current evaluation of the implementation of the co-designed InnoWell Platform (approved by the University of Sydney Human Research Ethics Committee, protocol number: 2018/962 [33]), which aims to determine how this technology solution is used to enhance Butterfly’s National Helpline.

### Limitations

A limitation of the research was that the workshops were not audio recorded. This was intentional, as dynamic individual and small group work took place making recording difficult, and to purposefully increase participant comfort and privacy. A trained scribe was presented to take detailed hand-written notes and quotes throughout the workshop, and facilitators also took notes that were triangulated for the workshop report; however, it is possible that some quotes are short-hand rather than verbatim. To value participant input, participants who did not participate during their working hours with the Butterfly’s National Helpline were also offered an Aus $50 (US $ 38.01) voucher for participating. Although this amount is higher than often provided in research, the Butterfly Foundation and the research team chose this amount as it was viewed as commensurate to the 3-hour time frame of the workshops and associated travel costs (particularly in regional areas).

As a final limitation, the findings presented in this research are only a prototype. Further research is needed to understand the acceptability and usability of the platform in the context of the Helpline. An impact evaluation is currently being undertaken to assess the real-world validity of the co-designed solution.

### Conclusions

The meaningful engagement of all end users from Butterfly’s National Helpline community via participatory design processes demonstrated that the principles of the InnoWell Platform align with the recommendations of the National Agenda, which call for the provision of web-based prevention, early access pathways, self-help, and recovery assistance [1]. This technology solution stemming from these participatory design workshops is not an end point. The knowledge translated information gathered through stakeholder engagement across urban and regional Australia will guide the ongoing development of the platform. Ultimately, impact evaluation will provide ongoing feedback to ensure that this is a high-quality, cost-effective, evidenced-based, person-centered service for everyone affected by eating disorders or body image issues across Australia.

## Appendix

Multimedia Appendix 1 User journey of a support person.

## Abbreviations

HP: health professional
LE: lived experience
PBNS: participant background not specified
R&D: research and development
SO: supportive other

## References

[1] (2018). The National Agenda for Disorders 2017 to 2022. The Butterfly Foundation.

[2] Hayes P (2019). Millions for research into eating disorders.

[3] The Butterfly Foundation (2020). About Butterfly Foundation.

[4] Hickie IB, Davenport TA, Burns JM, Milton AC, Ospina-Pinillos Laura, Whittle L, Ricci CS, McLoughlin LT, Mendoza J, Cross SP, Piper SE, Iorfino F, LaMonica HM (2019). Project Synergy: co-designing technology-enabled solutions for Australian mental health services reform. Med J Aust.

[5] Cross S, Hickie I (2017). Transdiagnostic stepped care in mental health. Public Health Res Pract.

[6] Hickie IB, Scott J, McGorry PD (2013). Clinical staging for mental disorders: a new development in diagnostic practice in mental health. Med J Aust.

[7] Bjerkan J, Hedlund M, Hellesø Ragnhild (2015). Patients' contribution to the development of a web-based plan for integrated care - a participatory design study. Inform Health Soc Care.

[8] Hagen P, Collin P, Metcalf A, Nicholas M, Rahilly K, Swainston N (2012). Participatory Design of evidence-based online youth mental health promotion, intervention and treatment.

[9] Clemensen J, Larsen SB, Kyng M, Kirkevold M (2007). Participatory design in health sciences: Using cooperative experimental methods in developing health services and computer technology. Qual Health Res.

[10] LaMonica HM, Davenport TA, Burns J, Cross S, Hodson S, Veitch J, Hickie IB (2019). Technology-Enabled Mental Health Service Reform for Open Arms - Veterans and Families Counselling: Participatory Design Study. JMIR Form Res.

[11] Heintz M, Law E, Soleimani S (2015). Paper or Pixel? Comparing Paper- and Tool-Based Participatory Design Approaches. Human-Computer Interaction – INTERACT 2015.

[12] Pablos-Mendez A, Shademani R (2006). Knowledge translation in global health. J Contin Educ Health Prof.

[13] Braun V, Clarke V (2006). Using thematic analysis in psychology. Qualitative Research in Psychology.

[14] Milton AC, Mullan B, Hunt C (2016). Information giving challenges and support strategies at the time of a mental health diagnosis: qualitative views from Australian health professionals. Soc Psychiatry Psychiatr Epidemiol.

[15] Milton AC, Mullan BA (2015). A qualitative exploration of service users' information needs and preferences when receiving a serious mental health diagnosis. Community Ment Health J.

[16] Ospina-Pinillos L, Davenport TA, Ricci CS, Milton AC, Scott EM, Hickie IB (2018). Developing a Mental Health eClinic to Improve Access to and Quality of Mental Health Care for Young People: Using Participatory Design as Research Methodologies. J Med Internet Res.

[17] Bucci S, Morris R, Berry K, Berry N, Haddock G, Barrowclough C, Lewis S, Edge D (2018). Early Psychosis Service User Views on Digital Technology: Qualitative Analysis. JMIR Ment Health.

[18] Ospina-Pinillos L, Davenport TA, Navarro-Mancilla AA, Cheng VWS, Cardozo Alarcón Andrés Camilo, Rangel AM, Rueda-Jaimes GE, Gomez-Restrepo C, Hickie IB (2020). Involving End Users in Adapting a Spanish Version of a Web-Based Mental Health Clinic for Young People in Colombia: Exploratory Study Using Participatory Design Methodologies. JMIR Ment Health.

[19] McLachlan R, Gilfillan G, Gordon J (2013). Deep and persistent disadvantage in Australia.

[20] Vinson T, Rawsthorne M, Beavis A, Ericson M (2015). Dropping off the edge 2015: Persistent communal disadvantage in Australia.

[21] Australian Bureau of Statistics (2007). National Survey of Mental Health and Wellbeing: Summary of Results.

[22] Hunter E (2007). Disadvantage and discontent: A review of issues relevant to the mental health of rural and remote Indigenous Australians. Aust J Rural Health.

[23] Bishop L, Ransom A, Laverty M, Gale L (2017). Mental Health in Rural and Remote Communities.

[24] National Rural Health Alliance (2017). Mental health in rural and remote Australia. FACT SHEET - DECEMBER 2017.

[25] Aref-Adib G, O'Hanlon P, Fullarton K, Morant N, Sommerlad A, Johnson S, Osborn D (2016). A qualitative study of online mental health information seeking behaviour by those with psychosis. BMC Psychiatry.

[26] Hetrick SE, Robinson J, Burge E, Blandon R, Mobilio B, Rice SM, Simmons MB, Alvarez-Jimenez M, Goodrich S, Davey CG (2018). Youth Codesign of a Mobile Phone App to Facilitate Self-Monitoring and Management of Mood Symptoms in Young People With Major Depression, Suicidal Ideation, and Self-Harm. JMIR Ment Health.

[27] Juarascio AS, Goldstein SP, Manasse SM, Forman EM, Butryn ML (2015). Perceptions of the feasibility and acceptability of a smartphone application for the treatment of binge eating disorders: Qualitative feedback from a user population and clinicians. Int J Med Inform.

[28] Schmitt Z, Yarosh S (2018). Participatory Design of Technologies to Support Recovery from Substance Use Disorders. Proceedings of the ACM on Human-Computer Interaction.

[29] Davenport TA, LaMonica HM, Whittle L, English A, Iorfino F, Cross S, Hickie IB (2019). Validation of the InnoWell Platform: Protocol for a Clinical Trial. JMIR Res Protoc.

[30] Rowe SC, Davenport TA, Easton MA, Jackson TA, Melsness J, Ottavio A, Sinclair J, Hickie IB (2020). Co-designing the InnoWell Platform to deliver the right mental health care first time to regional youth. Aust J Rural Health.

[31] Monsenso (2017). The Monsenso mHealth solution for mental illnesses. Product sheet.

[32] Lauriks S, Buster MC, de Wit MA, Arah OA, Klazinga NS (2012). Performance indicators for public mental healthcare: a systematic international inventory. BMC Public Health.

[33] LaMonica H, Milton A, Braunstein K, Rowe S, Ottavio T, Jackson T, Easton M, Hambleton A, Hickie I, Davenport Tracey A (2020). Technology-Enabled Solutions for Australian Mental Health Services Reform: Impact Evaluation. JMIR Form Res.

