# Supplementary figures and images for: Technology-Enabled Reform in a Nontraditional Mental Health Service for Eating Disorders: Participatory Design Study
Source: J Med Internet Res. 2021 Feb 16;23(2):e19532. doi: 10.2196/19532 (PMC7925150; doi:10.2196/19532)

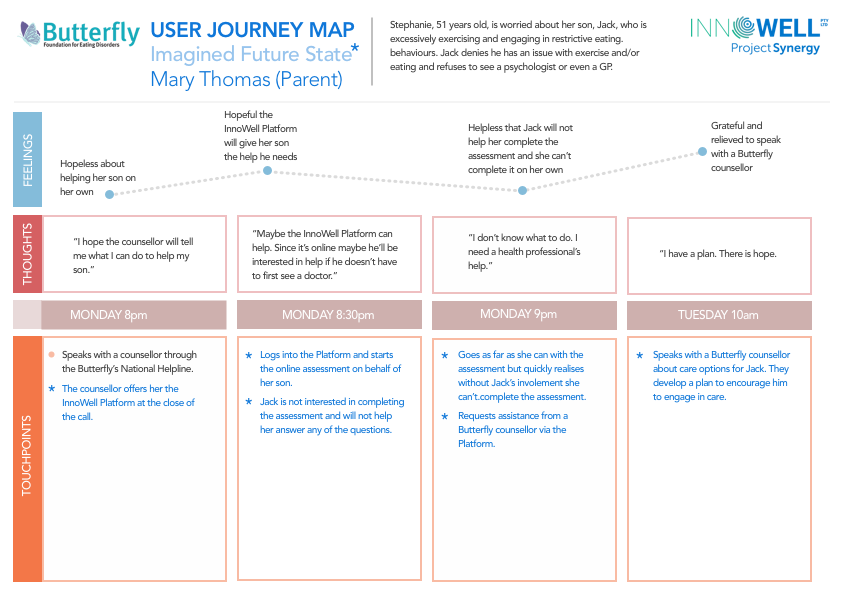

Supplement: Multimedia Appendix 1 [file jmir_v23i2e19532_app1.png]
